# Oral prehabilitation for patients with head and neck cancer: getting it right - the Restorative Dentistry-UK consensus on a multidisciplinary approach to oral and dental assessment and planning prior to cancer treatment

**DOI:** 10.1038/s41415-022-5197-3

**Published:** 2022-11-11

**Authors:** Lorna K. McCaul, Stewart Barclay, Peter Nixon, Pamela L. Yule, Jenna Trainor, Brian Stevenson, Andrew Paterson, Ailsa Nicol, Will Keys, Martin Donachie, Dean Barker, Sam Rollings, Simon Killough, James Ban, Andreas Chatzipantelis, Pallavi Gaitonde, Meena Ranka, Zaid Ali, Andrew MacInnes, Carly Taylor, Ashish Gopakumar, Praveen Sharma, Victoria Harper, Lakshmi Rasaratnam, Ishpinder Toor, Jose M. Rodriguez

**Affiliations:** 105510505085408940783 Consultant/Honorary Clinical Senior Lecturer in Restorative Dentistry, Glasgow Dental Hospital and School, 378 Sauchiehall Street, Glasgow, G2 3JZ, United Kingdom; 675458092099781192399 Honorary Secretary, Faculty of Dental Surgery, Royal College of Surgeons of Edinburgh, United Kingdom; 235763508352503515337 Consultant in Restorative Dentistry, York and Scarborough NHS Trust, UK; Visiting Associate Professor, University of Leeds, United Kingdom; 701377435467823325001 Consultant in Restorative Dentistry, Newcastle Dental Hospital, Richardson Road, Newcastle upon Tyne, NE2 4AZ, United Kingdom; 149678457989152721527 Consultant in Restorative Dentistry, The Newcastle upon Tyne Hospitals NHS Foundation Trust, Richardson Road, Newcastle upon Tyne, NE2 4AZ, United Kingdom; 048825747776736811129 Consultant in Restorative Dentistry, Dundee Dental Hospital and School, Park Place, Dundee, DD1 4HR, United Kingdom; 999814349937048260437 Clinical Senior Lecturer and Honorary Consultant in Restorative Dentistry, Glasgow Dental Hospital and School, 378 Sauchiehall Street, Glasgow, G2 3JZ, United Kingdom; 546837308569003668863 Consultant in Restorative Dentistry, Edinburgh Dental Institute, 39 Lauriston Place, Edinburgh, EH3 9HA, United Kingdom; 551800770916778789655 Consultant in Restorative Dentistry, Department of Restorative Dentistry, Aberdeen Dental Hospital and Institute of Dentistry, Cornhill Road, Aberdeen, AB25 2ZR, United Kingdom; 511619278454468833728 Consultant and Honorary Clinical Teacher in Restorative Dentistry, Department of Restorative Dentistry, Aberdeen Dental Hospital and Institute of Dentistry, Cornhill Road, Aberdeen, AB25 2ZR, United Kingdom; 986297309079157514044 Consultant in Restorative Dentistry, Belfast HSC Trust, Grosvenor Road, Belfast, BT12 6BA, United Kingdom; 287747745342382808651 Consultant in Restorative Dentistry, Cwm Taf Morgannwg University Health Board, Prince Charles Merthyr Tydfil, CF47 9DT, United Kingdom; 989326359697905784528 Speciality Registrar in Restorative Dentistry, Edinburgh Dental Institute, 39 Lauriston Place, Edinburgh, EH3 9HA, United Kingdom; 232625434065006536633 Consultant in Restorative Dentistry, Castle Hill Hospital, Hull and East Yorkshire NHS Trust, Cottingham, CH16 5JQ, United Kingdom; 614737504019235597586 Consultant in Restorative Dentistry, Luton and Dunstable University Hospital, Lewsey Road, Luton, LU4 0DZ, United Kingdom; 285833548638596841619 Consultant in Restorative Dentistry and Honorary Senior Lecturer, Leeds Dental Institute, The Worsley Building, Clarendon Way, Leeds, LS2 9LU, United Kingdom; 623185651597528323026 Consultant in Restorative Dentistry, Glasgow Dental Hospital and University Hospital, Crosshouse, Kilmarnock, KA2 0BE, United Kingdom; 351161419072395151946 Consultant/Honorary Senior Clinical Lecturer in Restorative Dentistry, University Dental Hospital of Manchester, Manchester University Foundation Trust, Higher Cambridge Street, Manchester, M15 6FH, United Kingdom; 241514371659662410920 Consultant in Restorative Dentistry, Luton and Dunstable Hospital, Lewsey Road, Luton, LU4 0DZ, United Kingdom; 535345704376566283891 Associate Professor and Honorary Consultant in Restorative Dentistry, University of Birmingham School of Dentistry, Birmingham, United Kingdom; 060220334247123024313 Clinical Senior Lecturer/Honorary Consultant in Restorative Dentistry, Glasgow Dental Hospital and School, 378 Sauchiehall Street, Glasgow, G2 3JZ, United Kingdom; 931930985288208278524 Consultant in Restorative Dentistry, East Kent Hospitals University Foundation Trust, United Kingdom; 326743955581259339171 Consultant in Restorative Dentistry, Guy´s and St Thomas´ NHS Foundation Trust, Guy´s Tower, Floor 26, Tower Wing, Great Maze Pond, London, SE1 9RT, United Kingdom; 629778065991578138793 Consultant in Restorative Dentistry and Honorary Clinical Senior Lecturer, Guy´s and St Thomas´ NHS Foundation Trust, Floor 26, Tower Wing, Great Maze Pond, SE1 9RT, United Kingdom

## Abstract

Historically, oral and dental issues for head and neck cancer patients were often not considered until after cancer treatment was complete. As a result, outcomes for oral rehabilitation were sometimes suboptimal. Inconsistencies in service delivery models and qualification, training and experience of staff delivering dental care often compounded this problem, making research and audit almost impossible. Collaborative working by consultants in restorative dentistry from all over the UK as part of a Restorative Dentistry-UK (RD UK) subgroup, renamed more recently as the RD-UK Head and Neck Cancer Clinical Excellence Network (CEN), has re-emphasised the importance of specialist restorative dentistry intervention at the outset of the head and neck cancer pathway to optimise outcomes of patient care. The CEN has driven several initiatives, reflecting Getting It Right First Time (GIRFT) principles aimed at reducing unwarranted variation. This improved consistency in approach and optimised collaborative working of the team now presents a better environment for multicentre audit and research. Ultimately, this should result in a continued improvement in patient and carer experience.

## Key points

- Optimal management of oral and dental care in the pre-treatment phase of the head and neck cancer pathway is key to producing the best possible outcomes for patients.
- Restorative dentistry consultants are core members of the head and neck cancer multidisciplinary team.
- The development of the Restorative Dentistry-UK Head and Neck Cancer Clinical Excellence Network has facilitated the standardisation of evidence-based pathways and models of care. It also provides support for all restorative dentistry consultants working in head and neck cancer, particularly those working single-handedly in district general hospitals.

## Background

### The impact of treatment for head and neck cancer on patients' oral and dental health

Approximately 11,500 patients in the UK are diagnosed with Head and Neck cancer (HNC) each year.^1^ These patients are referred to specialist HNC multidisciplinary teams (MDTs) for investigations and treatment, which may include surgery, radiotherapy and chemotherapy in various combinations. These treatment strategies can have a significant and long-lasting negative impact on oral and dental health, function and appearance.^2^ This, in turn, may have a detrimental effect on the patient's quality of life and psychological wellbeing.

Predicting and managing these issues is complex and requires close interaction with all members of the HNC MDT. The aim of an MDT should be to provide a collaborative, multi-professional environment facilitating effective care. Therefore, every MDT should have a consultant in restorative dentistry as a core member of the team.^3^^,^^4^^,^^5^^,^^6^^,^^7^ Their early involvement from the first MDT discussion is essential to ensure optimal outcomes at every stage of the pathway. The overall aim of the treatment strategy is to deliver high-quality, safe and effective person-centred care for patients in a timely manner, while ensuring services are optimised to offer responsive and multi-professional care. Evidence shows that patients' number one concern is survival, with their second most pressing concern being oral and dental issues such as dry mouth and chewing ability.^8^ The long-term effects of treatment for HNC on oral and dental health can be extremely distressing for patients. Historically, unwarranted variation both in the dental personnel delivering this service and in the approach to service delivery in the pre-treatment phase of the pathway in the UK existed. Many MDTs were non-compliant with the requirement for having a restorative dentist as core team member.

This paper describes how consultants in restorative dentistry have worked collaboratively in recent years to reduce this disparity and aims to share an agreed standard approach to planning oral and dental care before surgery or radiotherapy for HNC.

### Restorative dentistry

Restorative dentistry is one of the 13 UK dental specialties registered by the General Dental Council. Consultants in restorative dentistry are hospital-based clinicians who manage and coordinate highly complex dental care beyond the scope of primary care. This is often in the context of MDTs, such as those for HNC, hypodontia and cleft lip and palate, and involves working with medical specialists and specialist allied health professionals (for example, dietitians and speech and language therapists). Specialist restorative dentistry training includes the acquisition of comprehensive knowledge and skills in delivering oral and dental care before, during and after surgical and non-surgical treatment for HNC.^9^ It covers the provision of obturators, planning, placement and restoration of dental implants, planning pre-radiotherapy extractions and management of adverse oro-facial and dental complications of HNC treatment.

Restorative dentistry is the only dental speciality in the UK where such management of patients with HNC is an integral part of the training curriculum. Further, training in restorative dentistry includes comprehensive training in the three restorative mono-specialties: prosthodontics, endodontics and periodontics, and thus confers the ability to provide and coordinate all necessary planning, treatment and management of the adverse oro-facial and dental complications of patients with HNC.

### The Getting It Right First Time national programme

The Getting It Right First Time (GIRFT) Programme in England aims to improve medical care within the NHS by addressing unwarranted variation in care, identifying changes that will help improve care and outcomes and deliver cost savings.^7^ This is achieved by sharing best practice, resulting in responsive, transparent and open clinical practices.

For example, data from the* Hospital dentistry GIRFT programme national speciality report* suggest substantial variation in how hospital dentistry is commissioned and provided across the NHS in England.^7^ Overall, hospital dentistry contributes 5% to the total provision of dental care in England and this includes provision of highly complex restorative dentistry services for patients with HNC.

The role of collaborative working with professional organisations and networks is more important than ever to ensure restorative dentistry delivers the best care, thereby achieving the strategic goals of GIRFT. The aim is to deliver effective care by providing the right treatment (facilitated by evidence-driven, clinical guidelines resulting in a reduction in unwarranted, wasteful, and/or harmful variation), in the right way (appropriately delivered by suitably trained and skilled clinicians), to the right person, at the right time and in the right place (delivered by appropriately commissioned, staffed and structured services).

### Restorative Dentistry-UK and the Head and Neck Cancer Clinical Excellence Network

Restorative Dentistry-UK (RD-UK) is a not-for-profit organisation of consultants and specialists in restorative dentistry and a core function of RD-UK is to host Clinical Excellence Networks (CENs) including HNC, cleft lip and palate and hypodontia. There is evidence that the quality of patient care and patient outcomes can be improved by developing clinical networks.^10^ Following some years of informal engagement, RD-UK formed the HNC CEN in 2018, which established a network of consultants and speciality trainees in restorative dentistry working in HNC MDTs across the UK. The aim of this CEN is to improve the quality of oral and dental care for patients with HNC using several quality improvement strategies. These include multi-centre research, guideline development, audit, teaching and training, local and national service development support and improvement of information provision for patients and their carers. The HNC CEN collaborates with the other MDT clinical specialties (ear, nose and throat [ENT]; maxillofacial surgery; plastic surgery; oncology; cancer specialist nurses; dietitians; and speech and language therapists), to develop meaningful outcomes which are relevant and mutually agreed upon by all. The HNC CEN seeks to collaborate with and influence health policymakers and the third sector, to continually raise the profile of and improve the management of HNC patients.

### Strategies for reducing unwarranted variation in the oral prehabilitation and rehabilitation pathway

#### Reducing unwarranted variation in dental service design and delivery in the HNC pathway

The unique, broad-based training required to become a restorative dentistry consultant equips the specialist to act both as maxillofacial prosthodontist and dental oncologist. Dental oncologist is a term gaining traction around the world to describe specialists in the oral effects of non-surgical treatment modalities for HNC i.e. chemotherapy and/or radiotherapy. This dual function ensures that all patients treated with single or multiple modalities are risk assessed and treatment planned from the start, with expectations for post-treatment oral rehabilitation considered at the outset. As such, a consultant in restorative dentistry is a core member of the MDT.

Restorative dentistry consultants provide oral and dental prehabilitation and rehabilitation by considering all relevant periodontal, endodontic and prosthodontic factors and taking into account planned surgery or radiotherapy and any likely consequences from this to provide an optimum outcome for patients. Carrying out pre-treatment assessment and formulating an oral-rehabilitation plan presents considerable challenges as the outcomes of cancer treatment for an individual patient are unknown. Thus, risk assessment for adverse oral and dental outcomes forms a significant part of this stage of management. Furthermore, the dual role is especially important in the pre-treatment phase, where there are significant time pressures to prepare patients optimally for cancer treatment without delay, balanced with the need to achieve the best possible aesthetic and functional outcomes so that quality of life is enhanced. As such, the broad specialist training pathway of restorative dentistry ensures all these factors are appropriately considered by a consultant attending an MDT.

The requirement to have restorative dentistry representation in every MDT was recognised in the national HNC guidance for England (National Institute for Health and Care Excellence [NICE] 2004), Wales (National Standards for Head and Neck Cancer Services 2005) and Scotland (Scottish Intercollegiate Guidelines Network 2006). At that time, dental assessments were undertaken by clinicians from a variety of backgrounds, including general dental practitioners, oral surgeons and dental core trainees.

RD-UK have, over the last 15 years, worked on raising the profile of restorative dentistry in the HNC MDT via establishing representation on HNC standards and audit committees, HNC guideline working groups and registering as a stakeholder with quality improvement bodies, such as NICE. The National HNC Audit in England, known as Data for Head and Neck Oncology (DAHNO), reported annually from 2006-2015. The first DAHNO report (2004-2005) showed that pre-treatment dental assessment was recorded for only 5% of patients with cancer in the oral cavity.^11^ At the time, there was no representative from restorative dentistry on the relevant National Clinical Intelligence Network HNC Clinical Reference Group, despite the speciality being identified as core to the MDT. Engagement by the specialty of Restorative Dentistry in subsequent years helped to improve data capture.

By the tenth DAHNO report (2013-2014) pre-treatment dental assessment was noted in 30% of patients which was still suboptimal but an improvement.^12^ The outcomes were attributed to an apparent lack of restorative dentists in MDTs and perhaps the quality of data collection.

The tenth report noted that the 'importance of these specialists as core members of an MDT is recognised in improving outcomes guidance and BAHNO standards'. It also noted, however, that 'pre-treatment dental assessment has remained a controversial measure both in its definition and with difference of opinion on its value'. Historically, oral health has perhaps not always been viewed by non-dental colleagues as integral to general health. Furthermore, the nuances in provision of care by each of the 13 dental specialties can, on occasion, be under-appreciated, so the oral and dental aspects of care have sometimes been underestimated.

Such unwarranted variation in clinical background, training and qualifications of the staff carrying out assessments in this highly complex and specialised area risks unnecessary extractions and missed opportunities for optimisation of future oral rehabilitation, such as primary dental implant placement. Additionally, if future risks of osteoradionecrosis (ORN), caries and periodontal disease are not fully considered, avoidable long-term complications, resulting in additional costs, morbidity and unnecessary distress for patients, going against the principles outlined in GIRFT are a likely outcome. Advocacy for inclusion of restorative dentistry in the MDT continued over the next few years.

In 2019, the RD-UK HNC CEN carried out an audit of HNC MDTs in the UK and reported 100% of MDTs in Scotland and Wales and 80% of MDTs in England had a consultant in restorative dentistry at the MDT, which was a substantial improvement on the early DAHNO reports. The network has supported some of the remaining non-compliant MDTs since this audit was carried out and compliance is now estimated to be nearer to 85%. At the time of writing, there is, unfortunately, no national HNC audit in England.

Attainment of 100% compliance within the whole UK is now within reach and variation should be further reduced by new restorative dentistry speciality training proposals. These include trainee experience in a district general hospital (DGH) setting being considered as a core requirement to ensure that consultants are appropriately trained to support patients in both dental hospital and non-dental hospital environments. The RD-UK HNC CEN already plays a role in supporting those in single-handed or DGH-based units. The GIRFT *Hospital dentistry report* noted the importance of the HNC CEN in providing such support. Thus, in time, patients should be able to access highly complex oral rehabilitation closer to their homes, where appropriate, and this is becoming more common with the use of telemedicine platforms, which allow remote multi-speciality input, including restorative dentistry.

#### Evidence-based practice

It was identified by the RD-UK around ten years ago that there was unwarranted variation in the approach to oral prehabilitation and rehabilitation for patients with HNC and that guidelines for a structured, comprehensive, pathway-based approach, specific to prehabilitation and rehabilitation for HNC patients treated surgically or non-surgically, were not available in the UK. UK guidelines and standards for HNC pathways had brief outlines of dentally related issues but it was recognised that a guideline detailing the oral prehabiliation and rehabilitation aspects of the pathway was needed. In 2016 a detailed oral and dental HNC pathway guideline was therefore co-produced by RD-UK, the British Association of Head and Neck Oncologists, ENT UK, The Royal College of Speech and Language Therapists, The British Association of Head and Neck Oncology Nurses, and the British Dietetics Association This was endorsed by Royal College of Surgeons of England and the British Association of Head and Neck Oncologists.^6^ These guidelines have resulted in standardisation of care and better recognition of the core role of restorative dentistry in HNC in the UK. The RD-UK HNC CEN was also invited by ENT UK to contribute to the forthcoming sixth edition of *Head and neck cancer: United Kingdom national multidisciplinary guidelines.*

#### Research and audit

The evidence base for best practice in oral prehabiliation and rehabilitation is relatively sparse and there remains a need for high-quality, multi-centred and multi-disciplinary research. The HNC CEN is committed to encouraging and being involved in such research to improve the evidence base for the benefit of future generations of HNC patients and specialists alike. To deliver best practice as part of holistic, person-centred care, future research and quality improvement programmes should be aimed at improving some of the more challenging and controversial areas of the dental pathway for patients with HNC and focusing on what matters most to patients. This includes improving the head and neck surgery/restorative dentistry interface at the pre-surgical planning phase to ensure prompt oral rehabilitation. The focus will also be on developing dentally focused patient-related outcome measures (PROMs), as PROMs are relevant to the commissioning of NHS services and grant funding. The emphasis, via the RD-UK HNC CEN, will be on developing clear research questions, with a multi-centred approach facilitated by national collaboration and coordination. The RD-UK CEN have developed patient information resources specific to oral and dental issues for HNC patients with the aim of providing consistent and easily accessible patient information at all stages of the HNC pathway.

National and international audits still lack in-depth data relating to dental outcomes, perhaps due to a previous lack of detailed guidelines and lack of standardised outcomes. Alongside continued development of national HNC audits, a focus on development of a restorative dentistry minimum dataset for audit data collection would help produce more meaningful audit outcomes. Such a dataset might include data on decayed, missing and filled teeth, trismus, xerostomia and masticatory function, so that risk-adjusted outcomes can be developed. It might also include data on dental implants, pre-operative extractions and complications and workforce data from various centres. All these aspects are part of the restorative dentistry scope of practice, so the RD-UK HNC CEN is well placed to drive consensus on minimum datasets to inform future audit and research.

In the future, the role of the consultant restorative dentist will continue to evolve as the oral impacts of emerging technologies, such as robotic surgery, immunotherapy and proton beam therapy, become more apparent.

## The RD-UK HNC consensus approach to pre-surgery and pre-radiotherapy restorative dentistry planning for patients with HNC

The RD-UK HNC guidelines *Predicting and managing oral and dental complications of surgical and non-surgical treatment for head and neck cancer: a clinical guideline* gives guidance on the provision of oral and dental care before, during and after treatment for HNC.^6^ In 2018, the HNC CEN identified the pre-treatment stage of the pathway as being particularly challenging as the extent and nature of adverse oral and dental effects in any individual patient are not known at this stage. Thus, it constitutes a clinical risk assessment and plan based on expertly anticipated but not yet realised outcomes of surgical and non-surgical treatment. The importance of cancer prehabilitation and its role in improving health outcomes is recognised.^13^ This concept can be applied to dental health for cancer patients. The prehabilitation and planning stage is of great importance as inadequate prehabilitation counselling and management can result in significant adverse psychological and dental impacts. Unnecessary future costs can be incurred both by cancer services and by patients themselves if preventable conditions are not managed and opportunities for early rehabilitation interventions are missed. Examples include the cost of treating preventable primary disease, avoidable inpatient admissions for implant rehabilitation and the cost of managing osteoradionecrosis (ORN).

At a consensus meeting of the CEN in November 2018, areas of good practice within existing service delivery throughout the UK were identified and a standard approach on how to apply published guidelines and standards to the pre-treatment stage of the pathway were agreed.^5^^,^^6^^,^^14^ This discussion formed the basis of a draft document. The outcome of this consensus and subsequent discussions is reported here as a narrative summary with supporting references. These are practical recommendations on the application of the established principles of oral and dental care for patients with HNC. Recommendations of the RD-UK HNC guidelines relating to pre-radiotherapy dental extractions are supported by recent research investigating expert opinion of the topic.^15^

### Pre-treatment pathway aim

The aim of the restorative dentistry consultant in the pre-treatment phase should be to formulate an appropriate dental plan informed by the MDT-agreed cancer treatment plan for surgical or non-surgical treatment. This includes evaluating current and future dental and oral needs and considering anticipated post-treatment anatomy and functional and aesthetic requirements of the patient, mouth, face and jaws. Standardised written patient information should be provided to ensure patients and carers can refer to it during and after treatment.

For majority of patients, HNC treatment will adversely affect their oral and dental function and/or appearance. They will require restorative dentistry pre-habilitation.^3^^,^^4^^,^^5^^,^^6^^,^^14^ This may include patients planned for surgical intervention that will alter oral and/or facial anatomy, patients requiring radiotherapy where the treatment field includes any part of the maxilla, mandible or salivary glands and patients with specific dental concerns or pre-existing conditions. Edentulous patients may have retained roots, buried teeth or local bony pathology. They require radiographic assessment and should also be considered by the MDT for pre-habilitation, particularly if post-surgical anatomy will render the patient unable to wear a conventional fixed or removable prosthesis, thereby necessitating the use of dental implants. This stage of the pathway is often referred to as 'screening' or 'assessment'. These terms are not recommended as they may be misunderstood as meaning a routine check for common dental diseases. They fail to capture adequately this highly complex planning and initial treatment stage with its attendant time pressures. The terms 'planning' or 'oral and dental prehabilitation' are recommended.

Treatment planning at this stage is based on the anticipated impact of the planned cancer treatment on oral and dental health. Evidence-based risk assessment for risk of developing post-treatment, long-term oral and dental complications (altered anatomy, trismus, hyposalivation, radiotherapy-associated caries and ORN) is carried out.

### MDT discussion stage

#### Restorative prehabilitation: patient selection and rationale

During the HNC MDT discussion, the restorative dentistry consultant should highlight any concerns and advocate for the patient in relation to their oral and dental health. Restorative prehabilitation can be challenging to coordinate as there is a narrow window of time between the surgical or non-surgical plan being made and cancer treatment commencing. Opportunities for timely dental intervention should be identified. Patients whose oral cavity, teeth, salivary glands and jaws will be affected by radiotherapy and/or chemotherapy covering the oropharynx, nasopharynx, maxilla, mandible, or parotid glands should have a comprehensive oral and dental assessment and an appropriate management plan made as early as possible after the cancer treatment plan is agreed. This will allow time for any necessary dental treatment and to ensure dental treatment planning is based on the implications of the MDT-agreed cancer treatment plan. Overall prognostic factors, informed by treatment intent, will drive an appropriate dental treatment strategy.

The prehabilitation stage should render the patient as dentally healthy as practicably possible before treatment and ensure the oral cavity can be maintained during treatment and rehabilitated after treatment. Patients who have had restorative dental planning before surgery will need further assessment and planning if adjuvant (chemo)radiotherapy is prescribed following MDT HNC discussion of the final histopathology outcome. In this case, it will be necessary to consider whether any dental extractions are necessary and whether any modifications are needed to the original plan considering the outcome of surgery. Where patients are planned for surgery which will alter the oral cavity causing altered anatomy, communications with the nasal cavity, trismus, or difficulties in oral access, a joint planning consultation with maxillofacial surgeons and restorative dentistry consultants is required. This is essential where maxillectomy procedures or primary dental implants are being planned.

Primary dental implants, placed at the same time as ablative resection procedures, should always be considered. The advantage being that the window of opportunity for implant placement is not missed where post-operative radiotherapy is later indicated. The disadvantage is that there is very little time and many variables (for example, the actual extent of the surgical resection and/or reconstruction) for implant position planning. Secondary implant placement allows more time for comprehensive planning and assessment of any post-treatment challenges but placement may be complicated due to altered anatomy, bulky flaps, reduced mouth opening and post-operative radiotherapy. Furthermore, it extends the length of the oral rehabilitation pathway. Digital workflows based on restoratively driven planning can greatly facilitate the planning and execution of implant-based oro-facial rehabilitation, allowing condensed treatment completion.

### Restorative prehabilitation planning stage for patients receiving surgical or non-surgical treatment modalities

The procedures which should be included as part of the restorative prehabilitation planning stage are summarised in Table 1.

**Table 1:** Restorative prehabilitation planning

Surgery	(Chemo) radiotherapy
**History should include**	
Tumour-node-metastasis staging/human papillomavirus status Cancer treatment plan Presenting dental concerns Relevant medical history, including information about previous surgery or chemoradiotherapy to the head and neck in the case of adjuvant treatment or recurrence Social history including domestic situation, current and past employment status, and any other social cancer risk factors Smoking and alcohol history Current and future nutritional intake (in discussion with the specialist dietitian). Dental history including motivations, anxiety, and attitudes to treatment. Whether registered with a general dental practitioner	
**Extra-oral examination**	
Cervical lymph nodes Temporomandibular joints Muscles of mastication Measurement of mouth opening ability using inter-incisal measurement if dentate. This should be measured between the 21 and 31 (in mm) and index teeth noted, if the 21 and 31 are missing	
**Intra-oral examination**	
Soft tissues (lips, buccal mucosa, floor of mouth, ventral, dorsal, lateral tongue, hard and soft palate, oropharynx) Periodontal tissues (oral hygiene, periodontal probing depths, bleeding on probing, supra- and sub-gingival calculus, recession, mobility, furcation involvement) Dentition (teeth present, caries, tooth wear, presence and quality of restorations, occlusion) Static and dynamic occlusion. The presence and condition of any existing fixed or removable prostheses	
**Radiographic examination**	
Dental panoramic tomogram Periapical and bitewing radiographs (if appropriate) Use of diagnostic computerised tomography scan Cone-beam computed tomography (if appropriate)	
**Special investigations**	
Sensibility testing of selected teeth	Sensibility testing of selected teeth Salivary flow rates (where indicated)
**Dental diagnoses**	
Diagnoses should be stated and risk factors identified	
**Oral prehabilitation**	
**Surgery**	**(Chemo) radiotherapy**
Consider oro-facial, dental, functional and aesthetic impacts of treatment Discussion of options for prosthetic rehabilitation if needed Joint planning with head and neck surgeon to discuss planned surgical reconstruction and rehabilitation Consideration of primary implant placement Plan conventional prosthetic dental rehabilitation where indicated Plan implant-based prosthetic dental rehabilitation where indicated: Obtain digital surgical resection and reconstruction plan where indicated Impressions for study models Intra-oral optical scan or optical scan of models Digital workflow planning (with maxillofacial surgeon and reconstructive clinical scientist where appropriate) Fabrication of guide Clinical photographs Fabrication of surgical obturator and interim obturator impression trays for maxillectomy, even in case where reconstruction is planned Arrange for hygiene phase therapy if appropriate	Discussion with patient regarding oral, dental, functional, and aesthetic impacts of treatment Plans and options for prosthetic dental rehabilitation where indicated Risk assessment for trismus Risk assessment for xerostomia and its long-term effects Risk assessment for radiotherapy associated caries Risk assessment for ORN Instruction on jaw-stretching exercises Advice on caries prevention during acute phase of treatment including advice on cariogenic potential of oral nutritional supplements. Current recommended methods of caries prevention may not be tolerated for some patients during (chemo)radiotherapy due to acute toxicity Prescription of 1.1% sodium fluoride toothpaste for patients >16 years old Provision of bio-available calcium and phosphate paste (for example, Recaldent regimes to commence immediately following radiotherapy) Arrange for hygiene phase therapy if appropriate Arrange for urgent treatment of diseased but restorable teeth to render patient fit for radiotherapy Arrange for pre-radiotherapy extractions if deemed necessary Early restorative review on completion of treatment is indicated

## Extraction of teeth before HNC treatment

Dental extractions for patients with HNC can be a traumatic, highly emotive experience, on a background of a recent diagnosis of cancer. Pre-treatment extractions are planned based on risk assessment. There are no randomised controlled trials measuring complications of dental extractions before or during the initial phase of radiotherapy and its effect in preventing ORN. All these factors make this a particularly challenging aspect of the prehabilitation phase. Extraction of teeth, if indicated, should be organised as early as possible after the cancer treatment plan is known to maximise healing time and expedite the pathway. However, care should be taken to avoid unnecessary dental extractions, especially where the cancer treatment plan is not yet clarified, or when the long-term prognosis for the patient is poor, so radical extractions are avoided in order to give patients a better quality of life. Where multimodality treatment is definite or where gross dental pathology exists, extraction during primary surgery should be considered. Several factors need to be considered when balancing the complex decisions to extract teeth before treatment. Due to the brief window before primary chemotherapy and/or radiotherapy starts or between surgery and adjuvant chemotherapy and/or radiotherapy, there is limited time to extract teeth. There may be, however, risk of developing ORN or infection at healing extraction sites if insufficient time is allowed between extraction and start of treatment.^15^ For those having post-operative radiotherapy, extractions of teeth can be more challenging due to post-surgical trismus, making access to posterior teeth virtually impossible in some cases. Additionally, if the radiotherapy mask has been fitted, extraction of teeth may affect positioning and dose of radiotherapy, although this is unsupported by evidence and some centres do not think extraction following mask fit is relevant. The accepted recommendations for extractions of teeth are:

- Early restorative dentistry involvement/opinion is key
- Aim for mucosal coverage with primary closure when extracting impacted teeth
- Minimally traumatic extractions by experienced clinicians which avoid bone removal where possible is best
- Extractions should be carried out a minimum of 7-10 days before starting radiotherapy
- Timing of extractions in patients having neoadjuvant chemotherapy should be planned closely with the oncology team
- Decisions about extraction of third molar teeth should be discussed with the oral and maxillofacial surgery team
- If the long-term prognosis for a patient is uncertain, the benefits of dental extractions versus the potential negative functional, psychological and aesthetic impacts on the patient need to be considered carefully and discussed sensitively with the patient.

## Restorative dentistry post-treatment review

Once cancer treatment has been carried out, review should be arranged with the restorative dentistry consultant. For patients who have had radiotherapy, the restorative team will review the patient until they have recovered from acute toxicities of radiotherapy, have resumed normality of diet and can carry out oral self-care. They will then usually be discharged to primary dental care with an ongoing plan. For those who have had surgery and require prosthetic oral/maxillofacial rehabilitation, the restorative dentistry consultant will plan and often execute this phase of treatment, including first and/or second stage implant surgery where indicated.

## Conclusion

Treatment for HNC progresses at a fast pace after diagnosis, so it is essential that patients receive prompt clinical care without unnecessary delays. The importance of early and appropriate specialist dental input by the right clinician with the right experience is paramount to help expedite patient pathways, provide the appropriate risk assessments and plan for future rehabilitations. Restorative dentistry consultants can coordinate and provide all prehabiliation and rehabilitation aspects of care which are essential to ensure patient-based functional and aesthetic outcomes are delivered and to aid patient recovery and to ensure support before, during and after their journey. This input is vital for optimal treatment outcomes. The prehabilitation phase of care is complex and challenging, so close collaboration and integration across the speciality is important to share good clinical practice and ensure patients receive the highest possible standard of clinical care.

Restorative dentistry consultants have worked collaboratively over the last few years as a network and with external bodies and professional groups to improve patient care using a number of approaches. These include contribution to national and local service development, development of dentally focused guidelines and contribution to guidelines produced by other bodies, building a database of consultants working in HNC across the UK, responding as a group to ten-year cancer plans in England and Scotland and joining the newly formed HNC Coalition UK. This work should ensure that the oral and dental health of HNC patients remains a priority in healthcare provision.
